# Supplementary material for: The Activity Reaction Core and Plasticity of Metabolic Networks
Source: PLoS Comput Biol. 2005 Dec 16;1(7):e68. doi: 10.1371/journal.pcbi.0010068 (PMC1314881; doi:10.1371/journal.pcbi.0010068)
Supplement: Supplementary file 1 [file pcbi.0010068.sd001.doc]

**The activity reaction core and plasticity of metabolic networks**

**E. Almaas, Z.N. Oltvai and A.-L. Barabási**

**Supplementary Material**

**Contents:**

1. **Calculation of P-values**
2. **Error analysis of lethality data**
3. **Calculation of shortest paths**
4. **Metabolic core reactions and metabolic pathways**
5. **Construction of *E. coli* metabolic core diagram**
6. **Metabolic core reactions**
7. **List of metabolite abbreviations**

**1. Calculation of P-values:**

To estimate the statistical validity of our results, we calculate their P-values; measuring the strength of the null hypothesis. We do so either through analytical or numerical calculations. Generally, we calculate

$P=\sum_{x>a}^{b} p(x)$,	(1)

representing the cumulative probability that an event *x>a* will happen by chance.

**(a) P-value for the distribution of lethal reactions in the core**

We test the null hypothesis that the number of lethal reactions observed in the core is due to a random event by finding the total probability *P* that *x* lethal reactions out of *n* possible will be found in a core with *M* reactions when the total number of reactions is *N*. This probability is given by the hypergeometric distribution as:

$P=\sum_{x=a}^{b} \left(\begin{matrix} n \\ x \end{matrix}\right)\left(\begin{matrix} N-n \\ M-x \end{matrix}\right)/\left(\begin{matrix} N \\ M \end{matrix}\right)$,	(2)

where *a* is the actual number of lethal core reactions, and *b* is the smaller of *n* or *M*.

**(b) Connected core**

We test the null hypothesis that the metabolic core would be connected by chance. We cannot do this analytically, since the probability *P* depends on the actual network structure. Instead, we calculate this *P*-value numerically by randomly selecting *x* reactions, where *x* is the size of the actual core, from the full reaction set. Using a simple network-burning algorithm, we can then find the size of the biggest connected component in the randomly selected reaction set. The burning algorithm is simply implemented as follows: (1) select a node *i* among the *x* reactions. (2) recursively delete node *i* and all its neighbors until no further nodes can be reached. (3) Select a new node *j* among those remaining. We repeat this procedure a minimum of 1e6 times. An estimate for *P* is given by the fraction of times we found a connected component of equal or larger size than the actual core.

**(c) Evolutionary retention in the core**

We test the null hypothesis that the calculated evolutionary retention (ERI) of the metabolic core is a result of chance selection. Again, we need to resort to numerical calculations since the probability *P* depends on the actual ERI values of the reactions. We estimate *P* by randomly selecting *x* (the size of the core ) numbers from the set of ERI values before calculating their average value *<E>*. An estimate for *P* is now given by the number of times we find *<E>* greater or equal to the actual value out of 1e6 trials.

**(d) Half-life times of the core**

We test the null hypothesis that the average mRNA half-life time of the metabolic core is the result of chance selection. We need to estimate *P* numerically, and we repeat the approach given in **(c)**.

**Figure S1. Number of shortest paths.** We calculate the number of shortest paths as function of network size for the scale-free Barabási-Albert network model. We find that the average number of shortest paths increases faster than *N^2^*.

**2. Error analysis of lethality data:**

We simulate the effect of false-positive (F/P) and false-negative (F/N) determination of lethality as follows. (1) With probability *p*, we change the classification of every essential gene to non-essential to emulate an F/P determination in the data set. (2) We then calculate the average lethality for the metabolic core and the non-core. (3) We repeat steps (1) and (2) a total of 10,000 times to reach convergence in the average lethality calculation. Similarly, we simulate of the effect of F/N’s in the data set by, in step (1), changing every non-essential gene into essential with a probability *p*. Our results are summarized in Table 1. For the simple situation where there is a one-to-one correspondence between genes and reactions, the lethality ratios will follow *r=x(1-p)* and *r=x(1-p)+p*, where *x* is the original ratio and *p* is the noise level, for F/P and F/N respectively. Comparing these expressions with the entries in Table 1, we find a good correspondence.

|  |  | *E. coli* |  |  |  | *S. cerevisiae* |  |  |
| --- | --- | --- | --- | --- | --- | --- | --- | --- |
| Noise, *p* | F/P; core | F/P; non-core | F/N; core | F/N; non- core | F/P; core | F/P; non-core | F/N; core | F/N; non-core |
| 0.00 | 74.7% | 19.6% | 74.7% | 19.6% | 84.0% | 15.6% | 84.0% | 15.6% |
| 0.05 | 70.9% | 18.7% | 75.9% | 23.6% | 79.7% | 14.8% | 84.8% | 19.8% |
| 0.10 | 67.3% | 17.7% | 77.3% | 27.7% | 75.8% | 14.0% | 85.6% | 24.0% |
| 0.15 | 63.5% | 16.7% | 78.5% | 31.7% | 71.3% | 13.2% | 86.4% | 28.2% |
| 0.20 | 59.8% | 15.7% | 79.8% | 35.7% | 67.1% | 12.5% | 87.2% | 32.5% |
| 0.25 | 56.0% | 14.7% | 81.0% | 39.7% | 62.9% | 11.7% | 88.0% | 36.7% |

**Table 1:** Numerical simulation of noise in the lethality data. F/P (false-positive) and F/N (false-negative) designate if the experimental data respectively overestimate or underestimate the occurrence of lethal genes.

**3. Calculation of shortest paths:**

We estimate the dependence of shortest paths on the size of a network by investigating the model of Barabási and Albert [1]. Growing the network according to the prescription for preferential attachment, we generate systems of size 200 to 102,400. We calculate the average number of all shortest paths by use of the breadth first search (BFS) algorithm from a randomly selected node in the network. Note that the BFS algorithm is capable of finding *all* the shortest paths between any two nodes [2]. This is repeated for 1000 realizations of each network size. We find that the average number of shortest paths increases faster than *N^2^* (see Figure S1).

**4. Metabolic core reactions and metabolic pathways:**

We investigated the membership of the metabolic core reactions in the various metabolic pathways. Of the core reactions, *E. coli* has 11 in the Glycolysis pathway, 7 in the Aromatic Amino Acid pathway, 7 in the salvage pathway, 15 in Membrane Lipid Biosynthesis, 9 in Riboflavin Biosynthesis (complete), 8 in Folate Biosynthesis (almost complete), 5 in Coenzyme A pathway, 19 in Cell Envelope Biosynthesis (almost complete), and 10 in the Peptidoglycan Biosynthesis pathway (complete). The remaining reactions are scattered over the metabolic network. For *S. cerevisiae*, we find 11 in the Cytosolic Fatty Acid Biosynthesis, 8 in Phospholipid Biosynthesis, 4 in the salvage pathways, and 6 in the Coenzyme A Biosynthesis. The rest are scattered over the metabolic network.

**5. Construction of *E. coli* metabolic core diagram:**

From the list of metabolic core reactions (see section 5) we constructed the diagram of the core metabolic network (Figure 2). For each reaction, we omitted cofactors like CoA and ATP for the sake of clarity. However, we kept all the substrates and products for the nucleoside-diphosphate, the cytidylate and the dTMP kinase reactions. We colored blue the metabolites that directly participate in the biomass production reaction, and links (enzymatic reactions) found to be lethal during LB conditions are colored red. The arrows on the links indicate a directed reaction. Links without arrows are capable of both positive and negative flux flow. To facilitate a simple layout, we had to repeat several metabolites in the diagram, and the dotted blue lines serve as guides to the eye for their duplicate placement.

**6. Metabolic core reactions:**

We list the metabolic core reactions for the bacteria *H. pylori* and *E. coli* and the yeast *S. cerevisiae* in the supplementary Excel-formatted file. This file contains the core reaction names, gene names, enzyme classification numbers and a reaction description, when available. For *E. coli*, we have colored the lethal reactions red.

**7. List of metabolite abbreviations:**

In the following, we give the complete list of the metabolite abbreviations used in the metabolic *in silico* model for *E. coli* and *H. pylori*. This is followed by a list of the metabolite abbreviations used in the model for *S. cerevisiae*.

| **Abbreviation** | **Metabolite Name** | **Abbreviation** | **Metabolite Name** |
| --- | --- | --- | --- |
| Abbreviation | Metabolite Name | H2 | Hydrogen |
| 12PPD | 1,2-Propanediol | H2O2 | Hydrogen peroxide |
| 13DPG | 1,3-bis-Phosphoglycerate | H2S | Hydrogen sulfide |
| 23DHB | 2,3-Dihydroxybenzoate | H2SO3 | Sulfite |
| 23DHBA | 2,3-Dihydroxybenzoyl-adenylate | HBA | 4-Hydroxy-benzyl-alcohol |
| 23DHDHB | 2,3-Dihydo-2,3-dihydroxybenzoate | HCYS | Homocysteine |
| 2A3O | 2-Amino-3-oxobutanoate | HEMEO | Heme O |
| 2KD6PG | 2-keto-3-deoxy-6-phospho-gluconate | HEXT | External H+ |
| 2O6H | 2-Octaprenol 6-hydroxyphenol | HIS | Histidine |
| 2OMHMB | 2-Octaprenyl-3-methyl-5-hydroxy-6-methoxy-1,4-benzoquinone | HISOL | Histidinol |
| 2OPMB | 2-Octaprenyl-6-methoxy-1,4-benzoquinone | HISOLP | L-Histidinol-phosphate |
| 2OPMMB | 2-Octaprenyl-3-methyl-6-methoxy-1,4-benzoquinone | HMB | Hydroxymethylbilane |
| 2OPMP | 2-Octaprenyl-6-methoxyphenol | HPHPYR | para-Hydroxy phenyl pyruvate |
| 2OPPP | 2-Octaprenylphenol | HSER | Homoserine |
| 2PG | 2-Phosphoglycerate | HYXN | Hypoxanthine |
| 3DDAH7P | 3-Deoxy-d-arabino heptulosonate-7-phosphate | ICHOR | Isochorismate |
| 3PG | 3-Phosphoglycerate | ICIT | Isocitrate |
| 3PSER | 3-Phosphoserine | IDP | Inosine diphosphate |
| 3PSME | 3-Phosphate-shikimate | IGP | Indole glycerol phosphate |
| 4HBZ | 4-Hydroxybenzoate | ILE | Isoleucine |
| 4HLT | 4-Hydroxy-l-tyrosine | IMACP | Imidazole acetyl-phosphate |
| 4PPNCYS | 4'-Phosphopantothenoylcysteine | IMP | Inosine monophosphate |
| 4PPNTE | 4'-Phosphopantetheine | INDOLE | Indole |
| 4PPNTO | 4'-Phosphopantothenate | INS | Inosine |
| 5MTA | 5-Methylthioadenosine | IPPMAL | 3-Isopropylmalate |
| 5MTR | 5-Methylthio-D-ribose | IPPP | Isopentyl pyrophosphate |
| 5MTR1P | 5-Methylthio-5-deoxy-D-ribulose 1-phosphate | ISUCC | a-Iminosuccinate |
| 5MTRP | S5-Methyl-5-thio-D-ribose 1-phosphate | ITP | Inosine triphosphate |
| A5P | D-Arabinose 5-phosphate | K | Potassium |
| A6RP | 5-Amino-6-ribitylamino-2,4(1H,3H)-pyrimidinedione | K2LIPIV | (KDO(2))-lipid IV(A) |
| A6RP5P | 5-Amino-6-(ribosylamino)-2,4-(1H,3H)-pyrimidinedione 5'-phosphate | KDO | 2-Dehydro-3-deoxy-D-octonate |
| A6RP5P2 | 5-Amino-2,6-dioxy-4-(5'-phosphoribitylamino)pyrimidine | KDOLIPIV | (KDO)-lipid IV(A) |
| AA | D-Alanyl-D-alanine | KDOP | 2-Dehydro-3-deoxy-D-octonate 8-phosphate |
| AACCOA | Acetoacetyl-CoA | KMB | a- keto-g-methiobutyrate |
| ABUT | 2-Aceto-2-hydroxy butyrate | LAC | D-Lactate |
| AC | Acetate | LACAL | Lactaldehyde |
| ACACP | Acetyl-ACP | LCTS | Lactose |
| ACAL | Acetaldehyde | LEU | Leucine |
| ACCOA | Acetyl-CoA | LIPA | (KDO(2))-lipid A (Endotoxin) |
| ACLAC | Acetolactate | LIPIV | Lipid IV(A) |
| ACP | acyl-carrier protein | LIPX | Lipid X |
| ACTP | Acetyl-phosphate | LLAC | L-Lactate |
| AD | Adenine | LLCT | L-Cystathionine |
| ADCHOR | 4-Amino-4-deoxychorismate | LPS | Lippolysaccharide |
| ADN | Adenosine | LRL5P | L-Ribulose-5-Phosphate |
| ADP | Adenosine diphosphate | LYS | L-Lysine |
| ADPGLC | From RBC | MAL | Malate |
| ADPHEP | ADP-Mannoheptose | MALACP | Malonyl-ACP |
| AGM | Agmatine | MALCOA | Malonyl-CoA |
| AHHMD | 2-Amino-4-hydroxy-6-hydroxymethyl dihydropteridine-pp | MAN1P | Mannose 1-Phosphate |
| AHHMP | 2-Amino-4-hydroxy-6-hydroxymethyl dihydropteridine | MAN6P | Mannose 6-Phosphate |
| AHM | 4-Amino-5-hydroxymethyl-2-methylpyrimidine | MDAP | Meso-diaminopimelate |
| AHMP | 4-Amino-5-hydroxymethyl-2-methylpyrimidine-phosphate | MELI | Melibiose |
| AHMPP | 4-Amino-5-hydroxymethyl-2-methylpyrimidine-pyrophosphate | MET | Methionine |
| AHTD | 2-Amino-4-hydroxy-6-(erythro-1-2-3-trihydroxypropyl) dihydropteridine-p | METHF | 5,10-Methenyl tetrahydrofolate |
| AICAR | 5-Phosphate-ribosyl-5-amino-4-imidazole carboxamide | METTHF | 5,10-Methylene tetrahydrofolate |
| AIR | 5-Phosphoribosyl-5-aminoimidazole | MK | Menaquinone |
| AKG | a-Ketoglutarate | MLT6P | Maltose 6-phosphate |
| AKP | a-Ketopantoate | MNT6P | Mannitol 6-Phosphate |
| ALA | Alanine | MTHF | 5-Methyl tetrahydrofolate |
| ALAV | D-Aminolevulinate | NA | Sodium |
| AMP | Adenosine monophosphate | NAAD | Nicotinic acid adenine dinucleotide |
| AN | Antranilate | NAARON | N-a-Acetyl ornithine |
| AONA | 8-Amino-7-oxononanoate | NAC | Nicotinate |
| APS | Adenylyl sulfate | NAD | Nicotinamide adenine dinucleotide |
| ARAB | Arabinose | NADH | Nicotinamide adenine dinucleotide reduced |
| ARG | Arginine | NADP | Nicotinamide adenine dinucleotide phosphate |
| ARGSUCC | L-Arginio succinate | NADPH | Dihydronicotinamide adenine dinucleotide phosphate reduced |
| ASER | O-Acetylserine | NAG | N-Acetylglucosamine |
| ASN | Asparagine | NAGLU | N-Acetyl glutamate |
| ASP | Aspartate | NAGLUSAL | N-Acetyl glutamate semialdehyde |
| ASPSA | Aspartic beta-semialdehyde | NAGLUYP | N-Acetyl glutamyl -phosphate |
| ASUC | Adenilsuccinate | NAGP | N-Acetylglucosamine 6-phosphate |
| ATP | Adenosine triphosphate | NAm | Nicotinamide |
| bALA | b-Alanine | NAMAN | N-Acetyl-D-mannosamine |
| BASP | b-Aspartyl phosphate | NAMN | Nicotinic acid mononucleotide |
| BCAA | Branched chain amino acid | NCAIR | 5'-Phosphoribosyl-5-carboxyaminoimidazole |
| bDG6P | b-D-Glucose 6-Phosphate | NH3 | Ammonia |
| bDGLC | b-D-Glucose | NMN | Nicotinamide mononucleotide |
| BT | Biotin | NPRAN | N-5-phosphoribosyl-antranilate |
| C120ACP | Dodecanoyl-[acyl-carrier protein] | NS26DP | N-Succinyl-l,l-2,6-diaminopimelate |
| C140 | Myristic acid | NS2A6O | N-Succinyl-2-amino-6-ketopimelate |
| C140ACP | Myristoyl-[acyl-carrier protein] | O2 | Oxygen |
| C141ACP | Tetradecenoyl-[acyl-carrier protein] | O4HBZ | 3-Octaprenyl-4-hydroxybenzoate |
| C160 | Palmitic acid | OA | Oxaloacetate |
| C160ACP | Hexadecanoyl-[acyl-carrier protein] | OBUT | Oxobutyrate or 2-ketobutyrate |
| C161ACP | Hexadecenoyl-[acyl-carrier protein] | OGT | Oxidized glutathione |
| C180 | Stearic acid | OHB | 3-Hydroxy-4-phospho-hydroxy-alpha-ketobutyrate |
| C181ACP | Oleoyl-[acyl-carrier protein] | OICAP | 2-Oxoisocaproate |
| CAASP | Carbamoyl aspartate | OIVAL | Oxoisovalerate |
| CADV | Cadaverine | OMP | Orotidylate |
| CAIR | 5-Phosphoribosyl-5-aminoimidazole-4-carboxylate | OMVAL | Oxomethylvalerate |
| CAP | Carbamoyl phosphate | OPEP | Oligopeptide |
| CBHCAP | 3-Carboxy-3-hydroxy-isocaproate | OPP | trans Octaprenyl pyrophosphate |
| CDP | Cytidine diphosphate | ORN | Ornithine |
| CDPDG | CDP-1,2-Diacylglycerol | OROA | Orotic acid |
| CDPETN | CDP-Ethanolamine | OSB | O-Succinylbenzoic acid |
| CHCOA | 6-Carboxyhexanoyl-coa | OSBCOA | O-Succinylbenzoyl-CoA |
| CHOR | Chorisimate | OSLHSER | O-Succinyl-l-homoserine |
| CIT | Citrate | OTHIO | Thioredoxin (oxidized form) |
| CITR | L-Citrulline | P5P | Pyridoxine-5'-phosphate |
| CL | Cardiolypin | PA | Phosphatidyl acid |
| CMP | Cytidine monophosphate | PABA | para-Aminobenzoic acid |
| CMPKDO | CMP-2-keto-3-deoxyoctanoate | PANT | Pantoate |
| CO2 | Carbon dioxide | PAP | Adenosine-3',5'-diphosphate |
| COA | Coenzyme A | PAPS | 3-Phosphoadenylyl sulfate |
| CPAD5P | 1-O-Carboxyphenylamino 1-deoxyribulose-5-phosphate | PBG | Probilinogen III |
| CPP | Coproporphyrinogen III | PC2 | Percorrin 2 |
| CTP | Cytidine triphosphate | PDLA | Pyridoxamine |
| CYS | Cysteine | PDLA5P | Pyridoxamine-5-phosphate |
| CYTD | Cytidine | PE | Phosphatidyl ethanolamine |
| CYTS | Cytosine | PEP | Phosphoenolpyruvate |
| D23PIC | 2,3-Dihydro dipicolinate | PEPT | Peptide |
| D26PIM | L,l-2,6-Diamino pimelate | PEPTIDO | Peptidoglycan |
| D6PGC | D-6-Phosphate-gluconate | PG | Phosphatidyl glycerol |
| D6PGL | D-6-Phosphate-glucono-delta-lactone | PGP | L-1-Phoshatidyl-glycerol-phosphate |
| D6RP5P | 2,5-Diamino-6-(ribosylamino)-4-(3H)-pyrimidinone 5'-phosphate | PHE | Phenylalanine |
| D8RL | 6,7-Dimethyl-8-(1-D-ribityl)lumazine | PHEN | Prephenate |
| DA | Deoxyadenosine | PHP | 3-Phosphohydroxypyruvate |
| DADP | Deoxyadenosine diphosphate | PHPYR | Phenyl pyruvate |
| DALA | D-Alanine | PHSER | O-Phospho-l-homoserine |
| DAMP | Deoxyadenosine monophosphate | PHT | Phospho-hydroxy-threonine |
| DANNA | 7,8-Diaminononanoate | PI | Phosphate (inorganic) |
| DATP | Deoxyadenosine triphosphate | PIP26DX | Delta-piperidine-2,6-dicarboxylate |
| DB4P | 3,4-Dihydroxy-2-butanone-4-phosphate | PL | Pyridoxal |
| DC | Deoxycytidine | PL5P | Pyridoxal 5'-phosphate |
| DCDP | Deoxycytidine diphosphate | PNTO | Pantothenate |
| DCMP | Deoxycytidine monophosphate | PPHG | Protoporphyrinogen |
| DCTP | Deoxycytidine triphosphate | PPI | Pyrophosphate |
| DG | Deoxyguanosine | PPIX | Protoporphyrin IX |
| DGDP | Deoxyguanosine diphosphate | PRAM | 5-Phosphate-b-D-ribosyl amine |
| DGLU | D-Glutamate | PRBAMP | Phosphoribosyl -AMP |
| DGMP | 2-Deoxy-guanosine-5-phosphate | PRBATP | Phosphoribosyl-ATP |
| DGR | D-1,2-Diacylglycerol | PRFICA | 5-Phosphate-ribosyl-formamido-4-imidazole carboxamide |
| DGTP | Deoxyguanosine triphosphate | PRFP | Phosphoribosyl-formimino-AICAR-phosphate |
| DHF | Dihydrofolate | PRLP | Phosphoribulosyl- formimino-AICAR-phosphate |
| DHMVA | 2,3-Dihydroxy-3-methyl-valerate | PRO | Proline |
| DHNA | 1,4-Dihydroxy-2-naphthoic acid | PRPP | Phosphoribosyl pyrophosphate |
| DHP | Dihydroneopterin | PS | Phosphatidyl serine |
| DHPP | Dihydroneopterin phosphate | PTH | Protoheme |
| DHPT | 7,8-Dihydropteroate | PTRC | Putrescine |
| DHSK | Dehydroshikimate | PYR | Pyruvate |
| DHVAL | Dihydroxy-isovalerate | PYRDX | Pyridoxine |
| DIMGP | D-Erythro imidazoleglycerol-phosphate | Q | Ubiquinone |
| DIN | Deoxyinosine | QA | Quinolinate |
| DIPEP | Dipeptide | QH2 | Ubiquinol |
| DISAC1P | 2,3-bis(3-hydroxytetradecanoyl)-D-glucosaminyl-1,6-beta-D-2,3-bis(3-hydroxytetradecanoyl)-beta-D-glucosaminyl 1-phosphate or dissacharide 1-P | R1P | Ribose 1-phosphate |
| DKMPP | 0 | R5P | Ribose 5-phosphate |
| DMK | Demethylmenaquinone | RBL | Ribulose |
| DMPP | Dimethylallyl pyrophosphate | RGT | Reduced glutathione |
| DOROA | Dihydroorotic acid | RIB | Ribose |
| DPCOA | Dephosphocoenzyme A | RIBFLV | Riboflavin |
| DQT | 3-Dehydroquinate | RL5P | D-Ribulose 5-phosphate |
| DR1P | Deoxyribose 1-Phosphate | RML | Rhamnulose |
| DR5P | Deoxyribose 5-Phosphate | RML1P | Rhamnulose 1-phosphate |
| DSAM | Decarboxylated adenosylmethionine | RMN | Rhamnose |
| DSER | D-Serine | RTHIO | Thioredoxin (reduced form) |
| DT | Thymidine | S7P | sedo-Heptulose 7-phosphate |
| DTB | Dethiobiotin | SAH | S-Adenosyl homocystine |
| DTDP | Thymidine diphosphate | SAICAR | 5-Phosphoribosyl-4-(N-succinocarboxyamide)-5-amino-imidazole |
| DTMP | Thymidine monophosphate | SAM | S-Adenosyl methionine |
| DTP | 1-Deoxy-d-threo-2-pentulose | SAMOB | S-Adenosyl-4-methylthio-2-oxobutanoate |
| DTTP | Thymidine triphosphate | SER | Serine |
| DU | Deoxyuridine | SERA | L-Seryl-adenylate |
| DUDP | Deoxyuridine diphosphate | SHCHC | 2-Succinyl-6-hydroxy-2,4-cyclohexadiene-1-carboxylate |
| DUMP | Deoxyuridine monophosphate | SHCL | Sirohydrochlorin |
| DUTP | Deoxyuridine triphosphate | SHEME | Siroheme |
| DX5P | 1-D-Deoxyxylulose-5-phosphate | SLA | Sialic acid |
| E4P | Erythrose 4-phosphate | SLF | Sulfate |
| ENTER | Enterochelin | SME | Shikimate |
| ER4P | Erythronate-4-phosphate | SME5P | Shikimate-5-phosphate |
| ETH | Ethanol | SPMD | Spermidine |
| F1P | Fructose 1-Phosphate | SSALTPP | Succinate semialdehyde - thiamine pyrophosphate |
| F6P | Fructose 6-phosphate | SUC6P | Surose 6-phosphate |
| FAD | Flavin adenine dinucleotide | SUCC | Succinate |
| FADH | Flavin adenine dinucleotide reduced | SUCCOA | Succinyl-CoA or Succinate CoA |
| FCL | Fuculose | SUCCSAL | Succinate semialdehyde |
| FCL1P | Fuculose 1-Phosphate | T3P1 | Glyceraldehyde 3-phosphate |
| FDP | Fructose 1,6-diphosphate | T3P2 | Dihydroxyacetone phosphate or glycerone phosphate |
| FGAM | 5-Phosphoribosyl-n-formylgycineamidine | TAG16P | Tagatose-1,6 diphosphate |
| FGAR | 5-Phosphoribosyl-n-formylglycineamide | TAG6P | Tagatose-6-phosphate |
| FMN | flavin mononucleotide | THF | Tetrahydrofolate |
| FOR | Formate | THIAMIN | Thiamin |
| FPP | trans, trans Farnesyl pyrophosphate | THMP | Thiamine-phosphate |
| FRU | Fructose | THR | Threonine |
| FTHF | 10-Formyltetrahydrofolate | THY | Thymine |
| FUC | Fucose | THZ | 4-Methyl-5-(beta-hydroxyethyl)thiazole |
| FUM | Fumarate | THZP | 4-Methyl-5-(beta-hydroxyethyl)thiazole phosphate |
| G1P | Glucose 1-phosphate | TPP | Thiamine-pyrophosphate |
| G6P | Glucose 6-phosphate | TRE6P | Trehalose 6-phosphate |
| GA1P | Glucosamine 1-phosphate | TRP | Tryptophan |
| GA6P | D-Glucosamine | TYR | Tyrosine |
| GABA | 4-Aminobutanoate | UDP | Uridine diphosphate |
| GABAL | g-aminobutyraldehyde | UDPG | UDP Glucose |
| GAL1P | Galactose 1-Phosphate | UDPG23A | UDP-2,3-bis(3-hydroxytetradecanoyl)glucosamine |
| GAR | 5-Phosphate-ribosyl glycineamide | UDPG2A | UDP-3-O-(3-hydroxytetradecanoyl)glucosamine |
| GC | L-g-Glutamylcysteine | UDPG2AA | UDP-3-O-(3-hydroxytetradecanoyl)-N-acetylglucosamine |
| GDP | Guanosine diphosphate | UDPGAL | UDP Galactose |
| GL | Glycerol | UDPNAG | UDP N-acetyl glucosamine |
| GL3P | Glycerol 3-phosphate | UDPNAGEP | UDP-N-acetyl-3-O-(1-carboxyvinyl)-D-glucosamine |
| GLAC | Galactose | UDPNAM | UDP-N-acetyl-D-muramate |
| GLAL | D-Glyceraldehyde | UDPNAMA | UDP-N-acetylmuramoyl-L-alanine |
| GLC | a-D-Glucose | UDPNAMAG | UDP-N-acetylmuramoyl-L-alanyl-D-glutamate |
| GLCN | Gluconate | UDPP | Undecaprenyl pyrophosphate |
| GLN | Glutamine | UMP | Uridine monophosphate |
| GLT6P | Glucitol 6-Phosphate | UNAGD | UDP-N-acetylmuramoyl-L-alanyl-D-glutamyl-meso-2,6-diaminoheptanedioate |
| GLTL1P | Galacitol-1-Phosphate | UNAGDA | UDP-N-acetylmuramoyl-L-alanyl-D-glutamyl-meso-2,6-diaminoheptanedioate- D-alanyl-D-alanine |
| GLU | Glutamate | UNPTDO | UDP-N-acetylmuramoyl-L-alanyl-D-glutamyl-meso-2,6-diaminoheptanedioate- D-alanyl-D-alanine- diphosphoundecaprenol |
| GLUGSAL | L-Glutamate g-semialdehyde | UPRG | Uroporphyrinogen III |
| GLUP | Glutamyl phosphate | URA | Uracil |
| GLX | Glyoxylate | UREA | Urea |
| GLY | Glycine | URI | Uridine |
| GLYCOGEN | Glycogen | UTP | Uridine triphosphate |
| GMP | Guanosine monophosphate | VAL | Valine |
| GN | Guanine | X5P | D-Xylulose-5-phosphate |
| GPP | trans Geranyl pyrophosphate | XAN | Xanthine |
| GSA | Glutamate-1-semialdehyde | XMP | Xantosine monophosphate |
| GSN | Guanosine | XTSN | Xanthosine |
| GTP | Guanosine triphosphate | XUL | D-Xylulose |
| GTRNA | L-Glutamyl-tRNA(glu) | XYL | D-Xylose |

The metabolite abbreviations used in the model for *S. cerevisiae* are as follows:

| **Abbreviation** | **Metabolite Name** | **Abbreviation** | **Metabolite Name** |
| --- | --- | --- | --- |
| 13GLUCAN | 1,3-beta-D-Glucan | H3MCOA | (S)-3-Hydroxy-3-methylglutaryl-CoA |
| 13PDG | 3-Phospho-D-glyceroyl phosphate | H3MCOAm | (S)-3-Hydroxy-3-methylglutaryl-CoAM |
| 23DAACP | 2,3-Dehydroacyl-[acyl-carrier-protein] | HACNm | But-1-ene-1,2,4-tricarboxylateM |
| 23PDG | 2,3-Bisphospho-D-glycerate | HACOA | (3S)-3-Hydroxyacyl-CoA |
| 2HDACP | Hexadecenoyl-[acp] | HAN | 3-Hydroxyanthranilate |
| 2MANPD | ("alpha"-D-mannosyl)(,2)-"beta"-D-mannosyl-diacetylchitobiosyldiphosphod olichol | HBA | 4-Hydroxy-benzyl alcohol |
| 2N6H | 2-Nonaprenyl-6-hydroxyphenol | HCIT | 2-Hydroxybutane-1,2,4-tricarboxylate |
| 2NMHMBm | 3-Demethylubiquinone-9M | HCITm | 2-Hydroxybutane-1,2,4-tricarboxylateM |
| 2NPMBm | 2-Nonaprenyl-6-methoxy-1,4-benzoquinoneM | HCYS | Homocysteine |
| 2NPMMBm | 2-Nonaprenyl-3-methyl-6-methoxy-1,4-benzoquinoneM | HEXT | H+EXT |
| 2NPMP | 2-Nonaprenyl-6-methoxyphenol | HHTRNA | L-Histidyl-tRNA(His) |
| 2NPMPm | 2-Nonaprenyl-6-methoxyphenolM | HICITm | HomoisocitrateM |
| 2NPPP | 2-Nonaprenylphenol | HIS | L-Histidine |
| 2PG | 2-Phospho-D-glycerate | HISOL | L-Histidinol |
| 3DDAH7P | 2-Dehydro-3-deoxy-D-arabino-heptonate 7-phosphate | HISOLP | L-Histidinol phosphate |
| 3HPACP | (3R)-3-Hydroxypalmitoyl-[acyl-carrier protein] | HKYN | 3-Hydroxykynurenine |
| 3PG | 3-Phospho-D-glycerate | Hm | H+M |
| 3PSER | 3-Phosphoserine | HMB | Hydroxymethylbilane |
| 3PSME | 5-O-(1-Carboxyvinyl)-3-phosphoshikimate | HOMOGEN | Homogentisate |
| 4HBZ | 4-Hydroxybenzoate | HPRO | trans-4-Hydroxy-L-proline |
| 4HLT | 4-Hydroxy-L-threonine | HSER | L-Homoserine |
| 4HPP | 3-(4-Hydroxyphenyl)pyruvate | HTRNA | tRNA(His) |
| 4PPNCYS | (R)-4'-Phosphopantothenoyl-L-cysteine | HYXN | Hypoxanthine |
| 4PPNTE | Pantetheine 4'-phosphate | IAC | Indole-3-acetate |
| 4PPNTEm | Pantetheine 4'-phosphateM | IAD | Indole-3-acetamide |
| 4PPNTO | D-4'-Phosphopantothenate | ICIT | Isocitrate |
| 5MTA | 5'-Methylthioadenosine | ICITm | IsocitrateM |
| 6DGLC | D-Gal alpha 1->6D-Glucose | IDP | IDP |
| A6RP | 5-Amino-6-ribitylamino-2,4 (1H, 3H)-pyrimidinedione | IDPm | IDPM |
| A6RP5P | 5-Amino-6-(5'-phosphoribosylamino)uracil | IGP | Indoleglycerol phosphate |
| A6RP5P2 | 5-Amino-6-(5'-phosphoribitylamino)uracil | IGST | 4,4-Dimethylcholesta-8,14,24-trienol |
| AACCOA | Acetoacetyl-CoA | IIMZYMST | Intermediate_Methylzymosterol_II |
| AACP | Acyl-[acyl-carrier-protein] | IIZYMST | Intermediate_Zymosterol_II |
| ABUTm | 2-Aceto-2-hydroxy butyrateM | ILE | L-Isoleucine |
| AC | Acetate | ILEm | L-IsoleucineM |
| ACACP | Acyl-[acyl-carrier protein] | IMACP | 3-(Imidazol-4-yl)-2-oxopropyl phosphate |
| ACACPm | Acyl-[acyl-carrier protein]M | IMP | IMP |
| ACAL | Acetaldehyde | IMZYMST | Intermediate_Methylzymosterol_I |
| ACALm | AcetaldehydeM | INAC | Indoleacetate |
| ACAR | O-Acetylcarnitine | INS | Inosine |
| ACARm | O-AcetylcarnitineM | IPC | Inositol phosphorylceramide |
| ACCOA | Acetyl-CoA | IPPMAL | 2-Isopropylmalate |
| ACCOAm | Acetyl-CoAM | IPPMALm | 2-IsopropylmalateM |
| ACLAC | 2-Acetolactate | IPPP | Isopentenyl diphosphate |
| ACLACm | 2-AcetolactateM | ISUCC | a-Iminosuccinate |
| ACm | AcetateM | ITCCOAm | Itaconyl-CoAM |
| ACNL | 3-Indoleacetonitrile | ITCm | ItaconateM |
| ACOA | Acyl-CoA | ITP | ITP |
| ACP | Acyl-carrier protein | ITPm | ITPM |
| ACPm | Acyl-carrier proteinM | IZYMST | Intermediate_Zymosterol_I |
| ACTAC | Acetoacetate | K | Potassium |
| ACTACm | AcetoacetateM | KYN | L-Kynurenine |
| ACYBUT | gamma-Amino-gamma-cyanobutanoate | LAC | (R)-Lactate |
| AD | Adenine | LACALm | (S)-LactaldehydeM |
| ADCHOR | 4-amino-4-deoxychorismate | LACm | (R)-LactateM |
| ADm | AdenineM | LCCA | a Long-chain carboxylic acid |
| ADN | Adenosine | LEU | L-Leucine |
| ADNm | AdenonsineM | LEUm | L-LeucineM |
| ADP | ADP | LGT | (R)-S-Lactoylglutathione |
| ADPm | ADPM | LGTm | (R)-S-LactoylglutathioneM |
| ADPRIB | ADPribose | LIPOm | LipoamideM |
| ADPRIBm | ADPriboseM | LLACm | (S)-LactateM |
| AGL3P | Acyl-sn-glycerol 3-phosphate | LLCT | L-Cystathionine |
| AHHMD | 2-Amino-7,8-dihydro-4-hydroxy-6-(diphosphooxymethyl)pteridine | LLTRNA | L-lysyl-tRNA(Lys) |
| AHHMP | 2-Amino-4-hydroxy-6-hydroxymethyl-7,8-dihydropteridine | LLTRNAm | L-lysyl-tRNA(Lys)M |
| AHM | 4-Amino-5-hydroxymethyl-2-methylpyrimidine | LNST | Lanosterol |
| AHMP | 4-Amino-2-methyl-5-phosphomethylpyrimidine | LTRNA | tRNA(Lys) |
| AHMPP | 2-Methyl-4-amino-5-hydroxymethylpyrimidine diphosphate | LTRNAm | tRNA(Lys)M |
| AHTD | 2-Amino-4-hydroxy-6-(erythro-1,2,3-trihydroxypropyl)-dihydropteridine triphosphate | LYS | L-Lysine |
| AICAR | 1-(5'-Phosphoribosyl)-5-amino-4-imidazolecarboxamide | LYSm | L-LysineM |
| AIR | Aminoimidazole ribotide | MACOA | 2-Methylprop-2-enoyl-CoA |
| AKA | 2-Oxoadipate | MAL | Malate |
| AKAm | 2-OxoadipateM | MALACP | Malonyl-[acyl-carrier protein] |
| AKG | 2-Oxoglutarate | MALACPm | Malonyl-[acyl-carrier protein]M |
| AKGm | 2-OxoglutarateM | MALCOA | Malonyl-CoA |
| AKP | 2-Dehydropantoate | MALm | MalateM |
| AKPm | 2-DehydropantoateM | MALT | Malonate |
| ALA | L-Alanine | MALTm | MalonateM |
| ALAGLY | R-S-Alanylglycine | MAN | alpha-D-Mannose |
| ALAm | L-AlanineM | MAN1P | alpha-D-Mannose 1-phosphate |
| ALAV | 5-Aminolevulinate | MAN2PD | beta-D-Mannosyldiacetylchitobiosyldiphosphodolichol |
| ALAVm | 5-AminolevulinateM | MAN6P | D-Mannose 6-phosphate |
| ALTRNA | L-Arginyl-tRNA(Arg) | MANNAN | Mannan |
| AM6SA | 2-Aminomuconate 6-semialdehyde | MELI | Melibiose |
| AMA | L-2-Aminoadipate | MELT | Melibiitol |
| AMASA | L-2-Aminoadipate 6-semialdehyde | MET | L-Methionine |
| AMG | Methyl-D-glucoside | METH | Methanethiol |
| AMP | AMP | METHF | 5,10-Methenyltetrahydrofolate |
| AMPm | AMPM | METHFm | 5,10-MethenyltetrahydrofolateM |
| AMUCO | 2-Aminomuconate | METTHF | 5,10-Methylenetetrahydrofolate |
| AN | Anthranilate | METTHFm | 5,10-MethylenetetrahydrofolateM |
| AONA | 8-Amino-7-oxononanoate | MHIS | N(pai)-Methyl-L-histidine |
| APEP | Nalpha-Acetylpeptide | MI | myo-Inositol |
| APROA | 3-Aminopropanal | MI1P | 1L-myo-Inositol 1-phosphate |
| APROP | alpha-Aminopropiononitrile | MIP2C | Inositol-mannose-P-inositol-P-ceramide |
| APRUT | N-Acetylputrescine | MIPC | Mannose-inositol-P-ceramide |
| APS | Adenylylsulfate | MLT | Maltose |
| ARAB | D-Arabinose | MMET | S-Methylmethionine |
| ARABLAC | D-Arabinono-1,4-lactone | MNT | D-Mannitol |
| ARG | L-Arginine | MNT6P | D-Mannitol 1-phosphate |
| ARGSUCC | N-(L-Arginino)succinate | MTHF | 5-Methyltetrahydrofolate |
| ASER | O-Acetyl-L-serine | MTHFm | 5-MethyltetrahydrofolateM |
| ASN | L-Asparagine | MTHGXL | Methylglyoxal |
| ASP | L-Aspartate | MTHN | Methane |
| ASPERMD | N1-Acetylspermidine | MTHNm | MethaneM |
| ASPm | L-AspartateM | MTHPTGLU | 5-Methyltetrahydropteroyltri-L-glutamate |
| ASPRM | N1-Acetylspermine | MTRNAm | L-Methionyl-tRNAM |
| ASPSA | L-Aspartate 4-semialdehyde | MVL | (R)-Mevalonate |
| ASPTRNA | L-Asparaginyl-tRNA(Asn) | MVLm | (R)-MevalonateM |
| ASPTRNAm | L-Asparaginyl-tRNA(Asn)M | MYOI | myo-Inositol |
| ASUC | N6-(1,2-Dicarboxyethyl)-AMP | MZYMST | 4-Methylzymsterol |
| AT3P2 | Acyldihydroxyacetone phosphate | N4HBZ | 3-Nonaprenyl-4-hydroxybenzoate |
| ATN | Allantoin | NA | Sodium |
| ATP | ATP | NAAD | Deamino-NAD+ |
| ATPm | ATPM | NAADm | Deamino-NAD+M |
| ATRNA | tRNA(Arg) | NAC | Nicotinate |
| ATRP | P1,P4-Bis(5'-adenosyl) tetraphosphate | NACm | NicotinateM |
| ATT | Allantoate | NAD | NAD+ |
| bALA | beta-Alanine | NADH | NADH |
| BASP | 4-Phospho-L-aspartate | NADHm | NADHM |
| bDG6P | beta-D-Glucose 6-phosphate | NADm | NAD+M |
| bDGLC | beta-D-Glucose | NADP | NADP+ |
| BIO | Biotin | NADPH | NADPH |
| BT | Biotin | NADPHm | NADPHM |
| C100ACP | Decanoyl-[acp] | NADPm | NADP+M |
| C120ACP | Dodecanoyl-[acyl-carrier protein] | NAG | N-Acetylglucosamine |
| C120ACPm | Dodecanoyl-[acyl-carrier protein]M | NAGA1P | N-Acetyl-D-glucosamine 1-phosphate |
| C140 | Myristic acid | NAGA6P | N-Acetyl-D-glucosamine 6-phosphate |
| C140ACP | Myristoyl-[acyl-carrier protein] | NAGLUm | N-Acetyl-L-glutamateM |
| C140ACPm | Myristoyl-[acyl-carrier protein]M | NAGLUPm | N-Acetyl-L-glutamate 5-phosphateM |
| C141ACP | Tetradecenoyl-[acyl-carrier protein] | NAGLUSm | N-Acetyl-L-glutamate 5-semialdehydeM |
| C141ACPm | Tetradecenoyl-[acyl-carrier protein]M | NAM | Nicotinamide |
| C160 | Palmitate | NAMm | NicotinamideM |
| C160ACP | Hexadecanoyl-[acp] | NAMN | Nicotinate D-ribonucleotide |
| C160ACPm | Hexadecanoyl-[acp]M | NAMNm | Nicotinate D-ribonucleotideM |
| C161 | 1-Hexadecene | NAORNm | N2-Acetyl-L-ornithineM |
| C161ACP | Palmitoyl-[acyl-carrier protein] | NH3 | NH3 |
| C161ACPm | Palmitoyl-[acyl-carrier protein]M | NH3m | NH3M |
| C16A | C16_aldehydes | NPP | all-trans-Nonaprenyl diphosphate |
| C180 | Stearate | NPRAN | N-(5-Phospho-D-ribosyl)anthranilate |
| C180ACP | Stearoyl-[acyl-carrier protein] | O2 | Oxygen |
| C180ACPm | Stearoyl-[acyl-carrier protein]M | O2m | OxygenM |
| C181 | 1-Octadecene | OA | Oxaloacetate |
| C181ACP | Oleoyl-[acyl-carrier protein] | OACOA | 3-Oxoacyl-CoA |
| C181ACPm | Oleoyl-[acyl-carrier protein]M | OAHSER | O-Acetyl-L-homoserine |
| C182ACP | Linolenoyl-[acyl-carrier protein] | OAm | OxaloacetateM |
| C182ACPm | Linolenoyl-[acyl-carrier protein]M | OBUT | 2-Oxobutanoate |
| CAASP | N-Carbamoyl-L-aspartate | OBUTm | 2-OxobutanoateM |
| CAIR | 1-(5-Phospho-D-ribosyl)-5-amino-4-imidazolecarboxylate | OFP | Oxidized flavoprotein |
| CALH | 2-(3-Carboxy-3-aminopropyl)-L-histidine | OGT | Oxidized glutathione |
| cAMP | 3',5'-Cyclic AMP | OHB | 2-Oxo-3-hydroxy-4-phosphobutanoate |
| CAP | Carbamoyl phosphate | OHm | HO-M |
| CAR | Carnitine | OICAP | 3-Carboxy-4-methyl-2-oxopentanoate |
| CARm | CarnitineM | OICAPm | 3-Carboxy-4-methyl-2-oxopentanoateM |
| CBHCAP | 3-Isopropylmalate | OIVAL | (R)-2-Oxoisovalerate |
| CBHCAPm | 3-IsopropylmalateM | OIVALm | (R)-2-OxoisovalerateM |
| cCMP | 3',5'-Cyclic CMP | OMP | Orotidine 5'-phosphate |
| cdAMP | 3',5'-Cyclic dAMP | OMVAL | 3-Methyl-2-oxobutanoate |
| CDP | CDP | OMVALm | 3-Methyl-2-oxobutanoateM |
| CDPCHO | CDPcholine | OPEP | Oligopeptide |
| CDPDG | CDPdiacylglycerol | ORN | L-Ornithine |
| CDPDGm | CDPdiacylglycerolM | ORNm | L-OrnithineM |
| CDPETN | CDPethanolamine | OROA | Orotate |
| CER2 | Ceramide-2 | OSLHSER | O-Succinyl-L-homoserine |
| CER3 | Ceramide-3 | OSUC | Oxalosuccinate |
| CGLY | Cys-Gly | OSUCm | OxalosuccinateM |
| cGMP | 3',5'-Cyclic GMP | OTHIO | Oxidized thioredoxin |
| CHCOA | 6-Carboxyhexanoyl-CoA | OTHIOm | Oxidized thioredoxinM |
| CHIT | Chitin | OXA | Oxaloglutarate |
| CHITO | Chitosan | OXAm | OxaloglutarateM |
| CHO | Choline | P5C | (S)-1-Pyrroline-5-carboxylate |
| CHOR | Chorismate | P5Cm | (S)-1-Pyrroline-5-carboxylateM |
| cIMP | 3',5'-Cyclic IMP | P5P | Pyridoxine phosphate |
| CIT | Citrate | PA | Phosphatidate |
| CITm | CitrateM | PABA | 4-Aminobenzoate |
| CITR | L-Citrulline | PAC | Phenylacetic acid |
| CLm | CardiolipinM | PAD | 2-Phenylacetamide |
| CMP | CMP | PALCOA | Palmitoyl-CoA |
| CMPm | CMPM | PAm | PhosphatidateM |
| CMUSA | 2-Amino-3-carboxymuconate semialdehyde | PANT | (R)-Pantoate |
| CO2 | CO2 | PANTm | (R)-PantoateM |
| CO2m | CO2M | PAP | Adenosine 3',5'-bisphosphate |
| COA | CoA | PAPS | 3'-Phosphoadenylylsulfate |
| COAm | CoAM | PBG | Porphobilinogen |
| CPAD5P | 1-(2-Carboxyphenylamino)-1-deoxy-D-ribulose 5-phosphate | PC | Phosphatidylcholine |
| CPP | Coproporphyrinogen | PC2 | Sirohydrochlorin |
| CTP | CTP | PCHO | Choline phosphate |
| CTPm | CTPM | PDLA | Pyridoxamine |
| CYS | L-Cysteine | PDLA5P | Pyridoxamine phosphate |
| CYTD | Cytidine | PDME | Phosphatidyl-N-dimethylethanolamine |
| CYTS | Cytosine | PE | Phosphatidylethanolamine |
| D45PI | 1-Phosphatidyl-D-myo-inositol 4,5-bisphosphate | PEm | PhosphatidylethanolamineM |
| D6PGC | 6-Phospho-D-gluconate | PEP | Phosphoenolpyruvate |
| D6PGL | D-Glucono-1,5-lactone 6-phosphate | PEPD | Peptide |
| D6RP5P | 2,5-Diamino-6-hydroxy-4-(5'-phosphoribosylamino)-pyrimidine | PEPm | PhosphoenolpyruvateM |
| D8RL | 6,7-Dimethyl-8-(1-D-ribityl)lumazine | PEPT | Peptide |
| DA | Deoxyadenosine | PETHM | Ethanolamine phosphate |
| DADP | dADP | PGm | PhosphatidylglycerolM |
| DAGLY | Diacylglycerol | PGPm | PhosphatidylglycerophosphateM |
| DAMP | dAMP | PHC | L-1-Pyrroline-3-hydroxy-5-carboxylate |
| DANNA | 7,8-Diaminononanoate | PHE | L-Phenylalanine |
| DAPRP | 1,3-Diaminopropane | PHEN | Prephenate |
| DATP | dATP | PHP | 3-Phosphonooxypyruvate |
| DB4P | L-3,4-Dihydroxy-2-butanone 4-phosphate | PHPYR | Phenylpyruvate |
| DC | Deoxycytidine | PHSER | O-Phospho-L-homoserine |
| DCDP | dCDP | PHSP | Phytosphingosine 1-phosphate |
| DCMP | dCMP | PHT | O-Phospho-4-hydroxy-L-threonine |
| DCTP | dCTP | PI | Orthophosphate |
| DFUC | alpha-D-Fucoside | PIm | OrthophosphateM |
| DG | Deoxyguanosine | PIME | Pimelic Acid |
| DGDP | dGDP | PINS | 1-Phosphatidyl-D-myo-inositol |
| DGMP | dGMP | PINS4P | 1-Phosphatidyl-1D-myo-inositol 4-phosphate |
| DGPP | Diacylglycerol pyrophosphate | PINSP | 1-Phosphatidyl-1D-myo-inositol 3-phosphate |
| DGTP | dGTP | PL | Pyridoxal |
| DHF | Dihydrofolate | PL5P | Pyridoxal phosphate |
| DHFm | DihydrofolateM | PMME | Phosphatidyl-N-methylethanolamine |
| DHMVAm | (R)-2,3-dihydroxy-3-methylbutanoateM | PMVL | (R)-5-Phosphomevalonate |
| DHP | 2-Amino-4-hydroxy-6-(D-erythro-1,2,3-trihydroxypropyl)-7,8-dihydropteridine | PNTO | (R)-Pantothenate |
| DHPP | Dihydroneopterin phosphate | PPHG | Protoporphyrinogen IX |
| DHPT | Dihydropteroate | PPHGm | Protoporphyrinogen IXM |
| DHSK | 3-Dehydroshikimate | PPI | Pyrophosphate |
| DHSP | Sphinganine 1-phosphate | PPIm | PyrophosphateM |
| DHSPH | 3-Dehydrosphinganine | PPIXm | ProtoporphyrinM |
| DHVALm | (R)-3-Hydroxy-3-methyl-2-oxobutanoateM | PPMAL | 2-Isopropylmaleate |
| DIMGP | D-erythro-1-(Imidazol-4-yl)glycerol 3-phosphate | PPMVL | (R)-5-Diphosphomevalonate |
| DIN | Deoxyinosine | PRAM | 5-Phosphoribosylamine |
| DIPEP | Dipeptide | PRBAMP | N1-(5-Phospho-D-ribosyl)-AMP |
| DMPP | Dimethylallyl diphosphate | PRBATP | N1-(5-Phospho-D-ribosyl)-ATP |
| DMZYMST | 4,4-Dimethylzymosterol | PRFICA | 1-(5'-Phosphoribosyl)-5-formamido-4-imidazolecarboxamide |
| DOL | Dolichol | PRFP | 5-(5-Phospho-D-ribosylaminoformimino)-1-(5-phosphoribosyl)-imidazole-4-carboxamide |
| DOLMANP | Dolichyl beta-D-mannosyl phosphate | PRLP | N-(5'-Phospho-D-1'-ribulosylformimino)-5-amino-1-(5"-phospho-D-ribosyl)-4-imidazolecarboxamide |
| DOLP | Dolichyl phosphate | PRO | L-Proline |
| DOROA | (S)-Dihydroorotate | PROm | L-ProlineM |
| DPCOA | Dephospho-CoA | PRPP | 5-Phospho-alpha-D-ribose 1-diphosphate |
| DPCOAm | Dephospho-CoAM | PRPPm | 5-Phospho-alpha-D-ribose 1-diphosphateM |
| DPTH | 2-[3-Carboxy-3-(methylammonio)propyl]-L-histidine | PS | Phosphatidylserine |
| DQT | 3-Dehydroquinate | PSm | PhosphatidylserineM |
| DR1P | Deoxy-ribose 1-phosphate | PSPH | Phytosphingosine |
| DR5P | 2-Deoxy-D-ribose 5-phosphate | PTHm | HemeM |
| DRIB | Deoxyribose | PTRSC | Putrescine |
| DSAM | S-Adenosylmethioninamine | PURI5P | Pseudouridine 5'-phosphate |
| DT | Thymidine | PYR | Pyruvate |
| DTB | Dethiobiotin | PYRDX | Pyridoxine |
| DTBm | DethiobiotinM | PYRm | PyruvateM |
| DTDP | dTDP | QA | Pyridine-2,3-dicarboxylate |
| DTMP | dTMP | QAm | Pyridine-2,3-dicarboxylateM |
| DTP | 1-Deoxy-d-threo-2-pentulose | QH2m | UbiquinolM |
| DTTP | dTTP | Qm | Ubiquinone-9M |
| DU | Deoxyuridine | R1P | D-Ribose 1-phosphate |
| DUDP | dUDP | R5P | D-Ribose 5-phosphate |
| DUMP | dUMP | RAF | Raffinose |
| DUTP | dUTP | RFP | Reduced flavoprotein |
| E4P | D-Erythrose 4-phosphate | RGT | Glutathione |
| EPM | Epimelibiose | RGTm | GlutathioneM |
| EPST | Episterol | RIB | D-Ribose |
| ERGOST | Ergosterol | RIBFLAV | Riboflavin |
| ERTEOL | Ergosta-5,7,22,24(28)-tetraenol | RIBFLAVm | RiboflavinM |
| ERTROL | Ergosta-5,7,24(28)-trienol | RIPm | alpha-D-Ribose 1-phosphateM |
| ETH | Ethanol | RL5P | D-Ribulose 5-phosphate |
| ETHm | EthanolM | RMN | D-Rhamnose |
| ETHM | Ethanolamine | RTHIO | Reduced thioredoxin |
| F1P | D-Fructose 1-phosphate | RTHIOm | Reduced thioredoxinM |
| F26P | D-Fructose 2,6-bisphosphate | S17P | Sedoheptulose 1,7-bisphosphate |
| F6P | beta-D-Fructose 6-phosphate | S23E | (S)-2,3-Epoxysqualene |
| FAD | FAD | S7P | Sedoheptulose 7-phosphate |
| FADH2m | FADH2M | SACP | N6-(L-1,3-Dicarboxypropyl)-L-lysine |
| FADm | FADM | SAH | S-Adenosyl-L-homocysteine |
| FALD | Formaldehyde | SAHm | S-Adenosyl-L-homocysteineM |
| FDP | beta-D-Fructose 1,6-bisphosphate | SAICAR | 1-(5'-Phosphoribosyl)-5-amino-4-(N-succinocarboxamide)-imidazole |
| FERIm | Ferricytochrome cM | SAM | S-Adenosyl-L-methionine |
| FEROm | Ferrocytochrome cM | SAMm | S-Adenosyl-L-methionineM |
| FEST | Fecosterol | SAMOB | S-Adenosyl-4-methylthio-2-oxobutanoate |
| FGAM | 2-(Formamido)-N1-(5'-phosphoribosyl)acetamidine | SAPm | S-AminomethyldihydrolipoylproteinM |
| FGAR | 5'-Phosphoribosyl-N-formylglycinamide | SER | L-Serine |
| FGT | S-Formylglutathione | SERm | L-SerineM |
| FKYN | L-Formylkynurenine | SLF | Sulfate |
| FMN | FMN | SLFm | SulfateM |
| FMNm | FMNM | SME | Shikimate |
| FMRNAm | N-Formylmethionyl-tRNAM | SME5P | Shikimate 3-phosphate |
| FOR | Formate | SOR | Sorbose |
| FORm | FormateM | SOT | D-Sorbitol |
| FPP | trans,trans-Farnesyl diphosphate | SPH | Sphinganine |
| FRU | D-Fructose | SPRM | Spermine |
| FTHF | 10-Formyltetrahydrofolate | SPRMD | Spermidine |
| FTHFm | 10-FormyltetrahydrofolateM | SQL | Squalene |
| FUACAC | 4-Fumarylacetoacetate | SUC | Sucrose |
| FUC | beta-D-Fucose | SUCC | Succinate |
| FUM | Fumarate | SUCCm | SuccinateM |
| FUMm | FumarateM | SUCCOAm | Succinyl-CoAM |
| G1P | D-Glucose 1-phosphate | SUCCSAL | Succinate semialdehyde |
| G6P | alpha-D-Glucose 6-phosphate | T3P1 | D-Glyceraldehyde 3-phosphate |
| GA1P | D-Glucosamine 1-phosphate | T3P2 | Glycerone phosphate |
| GA6P | D-Glucosamine 6-phosphate | T3P2m | Glycerone phosphateM |
| GABA | 4-Aminobutanoate | TAG16P | D-Tagatose 1,6-bisphosphate |
| GABAL | 4-Aminobutyraldehyde | TAG6P | D-Tagatose 6-phosphate |
| GABALm | 4-AminobutyraldehydeM | TAGLY | Triacylglycerol |
| GABAm | 4-AminobutanoateM | TCOA | Tetradecanoyl-CoA |
| GAL1P | D-Galactose 1-phosphate | TGLP | N-Tetradecanoylglycylpeptide |
| GAR | 5'-Phosphoribosylglycinamide | THF | Tetrahydrofolate |
| GBAD | 4-Guanidino-butanamide | THFG | Tetrahydrofolyl-[Glu](n) |
| GBAT | 4-Guanidino-butanoate | THFm | TetrahydrofolateM |
| GC | gamma-L-Glutamyl-L-cysteine | THIAMIN | Thiamin |
| GDP | GDP | THMP | Thiamin monophosphate |
| GDPm | GDPM | THPTGLU | Tetrahydropteroyltri-L-glutamate |
| GDPMAN | GDPmannose | THR | L-Threonine |
| GGL | Galactosylglycerol | THRm | L-ThreonineM |
| GL | Glycerol | THY | Thymine |
| GL3P | sn-Glycerol 3-phosphate | THZ | 5-(2-Hydroxyethyl)-4-methylthiazole |
| GL3Pm | sn-Glycerol 3-phosphateM | THZP | 4-Methyl-5-(2-phosphoethyl)-thiazole |
| GLAC | D-Galactose | TPI | D-myo-inositol 1,4,5-trisphosphate |
| GLACL | 1-alpha-D-Galactosyl-myo-inositol | TPP | Thiamin diphosphate |
| GLAL | Glycolaldehyde | TPPP | Thiamin triphosphate |
| GLAM | Glucosamine | TRE | alpha,alpha-Trehalose |
| GLC | alpha-D-Glucose | TRE6P | alpha,alpha'-Trehalose 6-phosphate |
| GLN | L-Glutamine | TRNA | tRNA |
| GLP | Glycylpeptide | TRNAm | tRNAM |
| GLT | L-Glucitol | TRP | L-Tryptophan |
| GLU | L-Glutamate | TRPm | L-TryptophanM |
| GLUGSAL | L-Glutamate 5-semialdehyde | TRPTRNAm | L-Tryptophanyl-tRNA(Trp)M |
| GLUGSALm | L-Glutamate 5-semialdehydeM | TYR | L-Tyrosine |
| GLUm | GlutamateM | UDP | UDP |
| GLUP | alpha-D-Glutamyl phosphate | UDPG | UDPglucose |
| GLX | Glyoxylate | UDPGAL | UDP-D-galactose |
| GLY | Glycine | UDPNAG | UDP-N-acetyl-D-galactosamine |
| GLYCOGEN | Glycogen | UDPP | Undecaprenyl diphosphate |
| GLYm | GlycineM | UGC | (-)-Ureidoglycolate |
| GLYN | Glycerone | UMP | UMP |
| GMP | GMP | UPRG | Uroporphyrinogen III |
| GN | Guanine | URA | Uracil |
| GNm | GuanineM | UREA | Urea |
| GPP | Geranyl diphosphate | UREAC | Urea-1-carboxylate |
| GSN | Guanosine | URI | Uridine |
| GSNm | GuanosineM | UTP | UTP |
| GTP | GTP | VAL | L-Valine |
| GTPm | GTPM | X5P | D-Xylose-5-phosphate |
| GTRNA | L-Glutamyl-tRNA(Glu) | XAN | Xanthine |
| GTRNAm | L-Glutamyl-tRNA(Glu)M | XMP | Xanthosine 5'-phosphate |
| GTRP | P1,P4-Bis(5'-guanosyl) tetraphosphate | XTSINE | Xanthosine |
| H2O2 | H2O2 | XUL | D-Xylulose |
| H2S | Hydrogen sulfide | XYL | D-Xylose |
| H2SO3 | Sulfite | ZYMST | Zymosterol |
